# Robust Formation Control for Multiple Underwater Vehicles

**DOI:** 10.3389/frobt.2019.00090

**Published:** 2019-09-24

**Authors:** Charalampos P. Bechlioulis, Fotis Giagkas, George C. Karras, Kostas J. Kyriakopoulos

**Affiliations:** ^1^School of Mechanical Engineering, National Technical University of Athens, Athens, Greece; ^2^University of Thessaly, Lamia, Greece

**Keywords:** autonomous underwater vehicles, multi-agent systems, distance-based formation, prescribed performance control, decentralized control

## Abstract

This paper addresses the distance-based formation control problem for multiple Autonomous Underwater Vehicles (AUVs) in a leader-follower architecture. The leading AUV is assigned a task to track a desired trajectory and the following AUVs try to set up a predefined formation structure by attaining specific distances among their neighboring AUVs, while avoiding collisions and enabling at the same time relative localization. More specifically, a decentralized control protocol of minimal complexity is proposed that achieves prescribed, arbitrarily fast and accurate formation establishment. The control signal of each vehicle is calculated based on the relative position of its neighbors and its own velocity only, which can be easily acquired by the onboard sensors without necessitating for explicit network communication. Finally, a realistic simulation study with five AUVs performing seabed scanning was conducted to clarify the approach and verify the theoretical findings of this work.

## 1. Introduction

The use of autonomous underwater vehicles has steadily grown during the last 20 years. Several activities related to the offshore industry, such as surveillance and inspection of underwater facilities, oceanography, seabed map building, search and rescue, marine resource exploitation and so on, have been enabled by underwater robotic vehicles (Griffiths, 2002; Fossen, 2011; Zeng et al., 2015). However, most of the aforementioned applications are complex, time critical and may impose high level requirements in terms of accuracy, dexterity as well as time of completion. Thus, such strict specifications are almost impossible to be satisfied using a stand-alone vehicle. Moreover, single vehicle operation increases significantly the risk of mission failure due to sensor or actuator faults.

**Figure 1:**
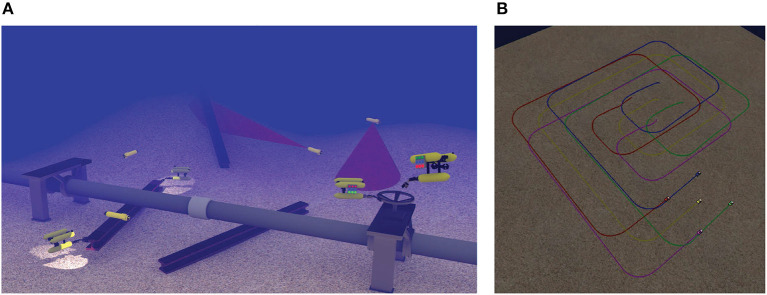
Examples of multiple underwater vehicles in cooperative missions. **(A)** Multiple vehicles cooperation for intervention tasks. **(B)** Multiple vehicles formation for surveillance tasks.

As an alternative solution, the deployment of multiple underwater vehicles in various formation schemes has emerged (see Figure 1). In this way, significant mission characteristics, such as completion time, fault tolerance, cognition and perception of the augmented system are positively affected. Numerous applications can benefit from the use of multiple underwater vehicles. An indicative example is the speed up process of map building for an environmental quantity (e.g., temperature, salinity) via en-route sampling of the water column (Caiti et al., 2007). Moreover, Simultaneous Localization and Mapping (SLAM) can be significantly improved, in terms of accuracy and speed, employing multiple underwater vehicles with complementary sensing and actuation capabilities (Walter and Leonard, 2004). Similarly, significantly better results can be accomplished in area patrolling for security or search and rescue purposes, where a usually large area should be thoroughly examined with increased confidence level (Kemp et al., 2004).

Another key feature of multi-agent systems is the upgrade of persistent autonomy via fault tolerance (Longhi et al., 2008). In most cases, AUVs are deployed for long periods of time (e.g., days or weeks) and their recovery in case of sensor or actuation faults is a costly and time consuming process. A proper formation re-planning can ensure the mission progress by allocating the faulty agents in non-critical but still important tasks. For example, in a surveillance and map building application, an agent with a faulty Multi-Beam Imaging Sonar (MBIS) can be excluded from the perception process, but still can act as a communication relay among the rest of the agents and the support vessel, by exploiting its acoustic communication equipment.

## 2. Related Work

Formation control, one of the most significant missions in underwater multi-agent systems, is a cooperative task in which multiple AUVs are deployed to achieve a specific geometric structure and move coordinately, so that a global mission is satisfied. Particularly in *leader-follower formation tracking*, a leading AUV aims at following a given trajectory that is defined by the mission goal and represents the required dynamic behavior of the group, while the followers are responsible for maintaining a desired formation, based on information related to their neighboring vehicles and the leader (Aguiar et al., 2007; Cui et al., 2008, 2012; Wang et al., 2009; Zhou et al., 2012a; Ma and Zeng, 2015). Alternatively, in *formation coordinated control* (Zhang and Qi, 2013; Zhao et al., 2016), the problem of steering a group of AUVs along certain paths and maintaining a desired rigid formation is tackled, while requesting limited communication among the agents. The dynamics of each AUV is considered known and is compensated by each controller locally whereas the coordination is achieved by adopting a distributed control law with limited information exchange among the vehicles (Ghabcheloo et al., 2006, 2009; Xiang et al., 2010). In *synchronized path following control* (Fan et al., 2010; Qi, 2014), the control protocols are decomposed in two modules that: (i) steer individually each AUV to trace predefined paths, and (ii) ensure that tracked paths are synchronized by distributed flocking under the constraints of multi-agent communication topology.

*Non-linear formation-keeping control* protocols for multiple AUVs have been proposed in Borhaug et al. (2007), Yang and Gu (2007), Cui et al. (2010), and Park (2015), where the formation is defined by the desired position and orientation of each follower with respect to its leader. Furthermore, finite-time consensus algorithms were proposed for both leaderless and leader-follower underwater multi-agent systems in Li and Wang (2013). Particularly in the leader-follower case, a distributed finite-time observer was developed to estimate the leader's velocity. Alternatively, *range-based formation control* was studied in Atta and Subudhi (2013) and Soares et al. (2013), where only range information with respect to the leader was incorporated in the control scheme. Similarly, in *hierarchical control* (Edwards et al., 2004; Ihue et al., 2004; Zhou et al., 2012b; Rout and Subudhi, 2016) the AUVs are equipped with heading detectors to achieve accurate relative localization. The leader is assigned a trajectory and is responsible for path tracking, maneuvering and guiding tasks, whereas the followers measure their distances and/or bearings to a set of neighboring agents to maintain the shape of a desired formation by keeping certain fixed distances. Finally, in a *passivity-based coordination* framework (Ihle et al., 2007; Jung et al., 2009; Wang et al., 2012), the desired formation patterns are obtained when the reference velocity assigned by a dynamic virtual leader is available to a subgroup of the AUVs.

Despite the recent progress in marine technology, the most significant challenge in underwater cooperation is imposed by the strict communication constraints, owing to the limited bandwidth and update rate of underwater acoustic devices. Furthermore, as the number of cooperating vehicles increases, communication protocols require complex design to deal with the crowded bandwidth (Stilwell and Bishop, 2000). Therefore, the number of underwater robots involved in cooperative schemes is strictly limited. Unfortunately, the majority of the aforementioned works in formation control necessitate for explicit communication among neighboring vehicles; thus suffering from the severe communication constraints that prohibit their implementation in real underwater missions. Contrary to the current state of the art, the proposed cooperative control protocol is purely decentralized and requires no underlying communication network to operate^1^. In particular, we propose a distance-based formation control protocol for a group of multiple AUVs in a leader-follower architecture, where the leader is assigned a task to track a desired trajectory and the followers try to establish a predefined formation structure by attaining specific distances among their neighboring AUVs, while avoiding collisions and enabling at the same time relative localization. The main contributions of this work can be summarized as follows.

1. *Decentralized Design:* The proposed design process is decentralized in the sense that each vehicle requires the relative position of its neighbors and its own velocity only, which can be easily acquired by the onboard sensors without necessitating for network communication.
2. *Reduced Complexity:* The proposed decentralized protocol requires simple calculations to output the control signal, thus it can be easily implemented on the embedded control systems of AUVs, and does not incorporate any prior knowledge of either the external disturbances or the vehicles' dynamic model parameters. Furthermore, no estimation has been employed to acquire such knowledge. Finally, the control protocol is independent of the global coordinate frame and does not require all local coordinate frames of the vehicles to be aligned.
3. *Robustness:* The actual transient and steady state response as well as the collision avoidance and relative localization specifications are determined a priori by the appropriate selection of certain performance functions and are isolated by the control gains selection (i.e., simple design) as well as the model uncertainties, extending thus greatly the robustness of the closed loop system.

The rest of the manuscript is organized as follows: section 3 introduces the problem, describes the system model and reviews preliminary results in rigid graphs. The control protocol along with the stability analysis are presented in section 4. Section 5 validates our approach via a simulated paradigm. Finally, section 6 concludes the paper and discusses future research directions.

## 3. Preliminaries

This section describes the dynamic model of the AUVs and introduces a rigorous formulation of the distance based formation control problem that will be tackled herein.

### 3.1. Vehicle Modeling

**Figure 2:**
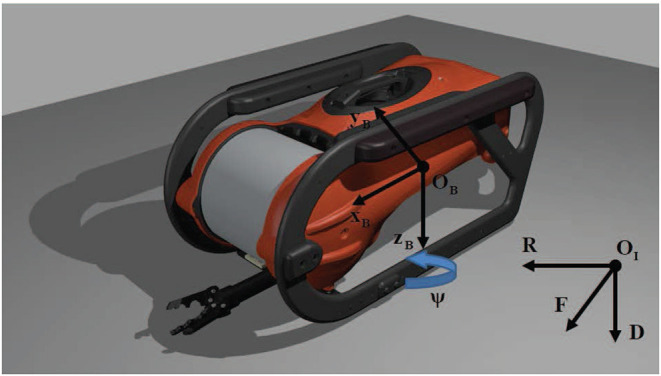
Inertial and body-fixed frames for an AUV model.

Let us define a body-fixed frame $B_{i}=\left\{e_{x},e_{y},e_{z}\right\}$ attached to the *i*-th vehicle's center of mass, as shown in Figure 2, and an inertial frame $I=\left\{e_{F},e_{R},e_{D}\right\}$ located at a fix position $O_{I}$ within the workspace. Moreover, assume that the vehicles behave as rigid bodies, the Earth rotation is negligible and the hydrodynamic coefficients remain constant. Thus, following standard modeling techniques (Fossen, 2011), the dynamic model of the vehicle in the body-fixed frame may be derived from the general Newton-Euler motion equations of a 6-DoFs rigid body subject to external forces and torques in a fluid medium, as follows:

(1) $$\begin{array}{l} M_{i}{\dot{\nu }}_{i}+C_{i}\left(\nu_{i}\right)\;\nu_{i}+D_{i}\left(\nu_{i}\right)\;\nu_{i}+g_{i}\left(\eta_{i}\right)=\tau_{E_{i}}+\tau_{i}\\ \;{\dot{\eta }}_{i}=J_{i}\left(\eta_{i}\right)\nu_{i} \end{array}$$

where:

- $\eta_{i}≜{\left[{\text{p}}_{i}^{T},{\text{q}}_{i}^{T}\right]}^{T}\in {ℜ}^{6}$ is the pose vector expressed in I, that involves the position (i.e., ${\text{p}}_{i}≜{\left[x_{i},y_{i},z_{i}\right]}^{T}$) and orientation (i.e., ${\text{q}}_{i}≜{\left[\phi_{i},\theta_{i},\psi_{i}\right]}^{T}$) vectors;
- $\nu_{i}≜{\left[{\text{v}}_{i}^{T},{\text{w}}_{i}^{T}\right]}^{T}\in {ℜ}^{6}$ is the velocity vector expressed in $B_{i}$, that involves the linear (i.e., ${\text{v}}_{i}≜{\left[u_{i},v_{i},w_{i}\right]}^{T}$) and angular (i.e., ${\text{w}}_{i}≜{\left[p_{i},q_{i},r_{i}\right]}^{T}$) velocity vectors;
- $\tau_{E_{i}}\in {ℜ}^{6}$ is the total environmental force/torque vector expressed in $B_{i}$, that is applied on the vehicle;
- $\tau_{i}≜{\left[\tau_{u_{i}},\tau_{v_{i}},\tau_{w_{i}},\tau_{p_{i}},\tau_{q_{i}},\tau_{r_{i}}\right]}^{T}\in {ℜ}^{6}$ is the total propulsion vector (i.e., the body forces τ_*u*_*i*__, τ_*v*_*i*__, τ_*w*_*i*__ and torques τ_*p*_*i*__, τ_*q*_*i*__, τ_*r*_*i*__ generated by the actuators) applied on the vehicle and expressed in $B_{i}$;
- **M**_*i*_ ≜ **M**_*RB*_*i*__ + **M**_*A*_*i*__, where ${\text{M}}_{RB_{i}}\in {ℜ}^{6\times 6}$ and ${\text{M}}_{A_{i}}\in {ℜ}^{6\times 6}$ are the rigid body and added mass inertia matrices, respectively;
- **C**_*i*_(ν_*i*_) ≜ **C**_*RB*_*i*__(ν_*i*_) + **C**_*A*_*i*__(ν_*i*_) , where ${\text{C}}_{RB_{i}}\left(\nu_{i}\right)\in {ℜ}^{6\times 6}$ and ${\text{C}}_{A_{i}}\left(\nu_{i}\right)\in {ℜ}^{6\times 6}$ are the rigid body and added mass matrices that model the Coriolis and centrifugal effects, respectively;
- **D**_*i*_(ν_*i*_) ≜ **D**_*q*_*i*__(ν_*i*_) + **D**_*l*_*i*__, where ${\text{D}}_{q_{i}}\left(\nu_{i}\right)\in {ℜ}^{6\times 6}$ and ${\text{D}}_{l_{i}}\in {ℜ}^{6\times 6}$ denote the quadratic and linear drag matrices, respectively;
- ${\text{g}}_{i}\left(\eta_{i}\right)\in {ℜ}^{6}$ is the gravity and hydrostatic restoring force vector;
- ${\text{J}}_{i}\left(\eta_{i}\right)≜\left[\begin{array}{cc} {\text{J}}_{i}^{\text{v}}\left({\text{q}}_{i}\right) & 0_{3\times 3}\\ 0_{3\times 3} & {\text{J}}_{i}^{\text{w}}\left({\text{q}}_{i}\right) \end{array}\right]$ is the Jacobian matrix transforming the velocities from the body-fixed frame $B_{i}$ to the inertial frame I, in which ${\text{J}}_{i}^{\text{v}}\left({\text{q}}_{i}\right)\in SO\left(3\right)$ stands for the rotation matrix and ${\text{J}}_{i}^{\text{w}}\left({\text{q}}_{i}\right)\in {ℜ}^{3\times 3}$ denotes the lumped transformation matrix (Fossen, 2011).

### 3.2. Coordination of Multiple AUVs

This work examines the coordination problem of *N* + 1 AUVs under a leader-follower architecture. The leader (indexed by 0) is assigned a desired trajectory (e.g., an exploratory task) for the multi-agent system and the *N* followers update their state using locally available feedback, which corresponds to measurements of the inter-agent distances/headings, i.e., we consider the *distance-based formation* control problem (Oh et al., 2015). Thus, each agent interacts with its neighbors in order to complete the assigned task. Herein, the interaction of the agents is modeled by undirected graphs. More specifically, we consider an undirected graph with *l* edges and *N* + 1 vertices (corresponding to the *N* + 1 AUVs of the multi-agent system), denoted by G≜(V,E) where V={0,1,…,N} is the set of vertices and E⊂V×V is the set of edges. The set of neighbors of the *i*-th AUV is defined as:

$$\begin{array}{l} N_{i}\left(E\right)=\left\{j\in V|\left(i,j\right)\in E\right\}. \end{array}$$

Moreover, ${\text{p}}_{i}\in {ℜ}^{3},i=0,1,\ldots ,N$ denotes the position of each AUV and the overall vector $\bar{\text{p}}≜\text{col}\left({\text{p}}_{i}\right)\in {ℜ}^{3\left(N+1\right)}$ represents the realization of G in ℜ^3^. The pair $F≜\left(G,\bar{\text{p}}\right)$ is said to be a *framework* of G. Since the sequence of edges in E is arbitrary, an edge function (rigidity function) $\Phi_{G}:{ℜ}^{3\left(N+1\right)}\to R^{l}$ associated with F may be given as :

(2) $$\begin{array}{r} \Phi_{G}\left(\bar{\text{p}}\right)={\left[\ldots ,∥{\text{p}}_{i}-{\text{p}}_{j}∥,\ldots \right]}^{T},\;\left(i,j\right)\in E. \end{array}$$

**Definition 1**. *The framework $F=\left(G,\bar{\text{p}}\right)$ is rigid at $\bar{\text{p}}\in {ℜ}^{3\left(N+1\right)}$ there exists a neighborhood $U_{\bar{\text{p}}}$ of $\bar{\text{p}}$ such that $\Phi_{G}^{-1}\left(\Phi_{G}\left(\bar{\text{p}}\right)\right)\cap U_{\bar{\text{p}}}=\Phi_{H}^{-1}\left(\Phi_{H}\left(\bar{\text{p}}\right)\right)\cap U_{\bar{\text{p}}}$, where H denotes the complete graph with N* + 1 *vertices and $\Phi_{⋆}^{-1}$ denotes the set of all points $\bar{\text{q}}\in {ℜ}^{3\left(N+1\right)}$ satisfying $\Phi_{⋆}\left(\bar{\text{p}}\right)=\Phi_{⋆}\left(\bar{\text{q}}\right)$ for any graph ⋆*.

This definition implies that in a rigid framework, keeping the edge length and at the same time moving one or a set of vertices of the graph does not affect the distances between the other vertices. Moreover, we define the *rigidity matrix* *R*:ℜ^3(*N* + 1)^ → ℜ^*l* × 3(*N* + 1)^ of $F=\left(G,\left(\bar{\text{p}}\right)\right)$ as:

(3) $$\begin{array}{r} R\left(\bar{\text{p}}\right)=\frac{\partial \Phi_{G}\left(\bar{\text{p}}\right)}{\partial \bar{\text{p}}}. \end{array}$$

Hence, given a sequence of edges in E, each row of the rigidity matrix $R\left(\bar{\text{p}}\right)$ takes the following form:

(4) $$\begin{array}{l} \left[0_{1\times 3}^{T},...,{\left(\frac{{\text{p}}_{i}-p_{j}}{\left||p_{i}-p_{j}|\right|}\right)}^{T},...,0_{1\times 3}^{T},...,{\left(\frac{p_{j}-p_{i}}{\left||p_{i}-p_{j}|\right|}\right)}^{T},...,0_{1\times 3}^{T}\right]\\ \in {ℜ}^{1\times 3(N+1)} \end{array}$$

Clearly, the rigidity matrix depends only on the relative positions, so it can be written as $R\left(\tilde{\text{p}}\right)$ where $\tilde{\text{p}}=\text{col}\left({\tilde{\text{p}}}_{ij}\right)\in {ℜ}^{3l}$ in which ${\tilde{\text{p}}}_{ij}={\text{p}}_{i}-{\text{p}}_{j},\left(i,j\right)\in E$.

**Definition 2**. *A framework $F=\left(G,\left(\bar{\text{p}}\right)\right)$ with *N* + 1 vertices is infinitesimally rigid in* ℜ^3^ *if:*

(5) $$\begin{array}{r} \text{rank}\left[R\left(\bar{\text{p}}\right)\right]=3\left(N+1\right)-6. \end{array}$$

It follows from the aforementioned definition that $F=\left(G,\bar{\text{p}}\right)$ is infinitesimally rigid in ℜ^3^ if the corresponding graph has at least 3(*N* + 1) − 6 edges, i.e., |E|≥3(N+1)-6.

**Definition 3**. *A rigid framework is said to be minimally rigid if no edge can be removed without causing the graph to lose its rigidity. In* ℜ^3^ *a rigid framework $\left(G,\left(\bar{\text{p}}\right)\right)$ is minimally rigid if |E|=3(N+1)-6*.

If a framework is infinitesimally rigid and its underlying graph has exactly 3(*N* + 1) − 6 edges, then it is called a *minimally and infinitesimally rigid* framework. If $\Phi_{G}\left(\bar{\text{p}}\right)=\Phi_{G}\left({\bar{\text{p}}}^{⋆}\right)$ applies to frameworks $\left(G,\bar{\text{p}}\right)$ and $\left(G,{\bar{\text{p}}}^{⋆}\right)$, these frameworks are said to be *equivalent*. Furthermore, if $∥{\text{p}}_{i}-{\text{p}}_{j}∥=∥{\text{p}}_{i}^{⋆}-{\text{p}}_{j}^{⋆}∥$ for ∀i,j∈V, then the two frameworks are *consistent*. Two infinitesimally rigid frameworks $\left(G,\bar{\text{p}}\right)$ and $\left(G,{\bar{\text{p}}}^{⋆}\right)$ are said to be *congruent* if they are equivalent but not consistent. Finally, the set Iso(F) denotes all isometric frameworks of F (i.e., all rotated, translated and reflected implementations).

**Lemma 1**. (Cai and De Queiroz, 2015) We consider two frameworks $F=\left(G,\bar{\text{p}}\right)$ and $F^{*}=\left(G,{\bar{\text{p}}}^{*}\right)$ which share the same graph G=(V,E). If $F^{*}$ is infinitesimally rigid and $\text{dist}\left(\bar{\text{p}};\text{Iso}\left(F^{*}\right)\right)\leq \epsilon$ where ϵ is a sufficient small positive constant, then F is also infinitesimally rigid.

**Lemma 2**. *(Cai and De Queiroz, 2015) If the framework $F=\left(G,\bar{\text{p}}\right)$ is minimally and infinitesimally rigid, then the matrix $R\left(\bar{\text{p}}\right)R{\left(\bar{\text{p}}\right)}^{T}$ is positive definite*.

**Figure 3:**
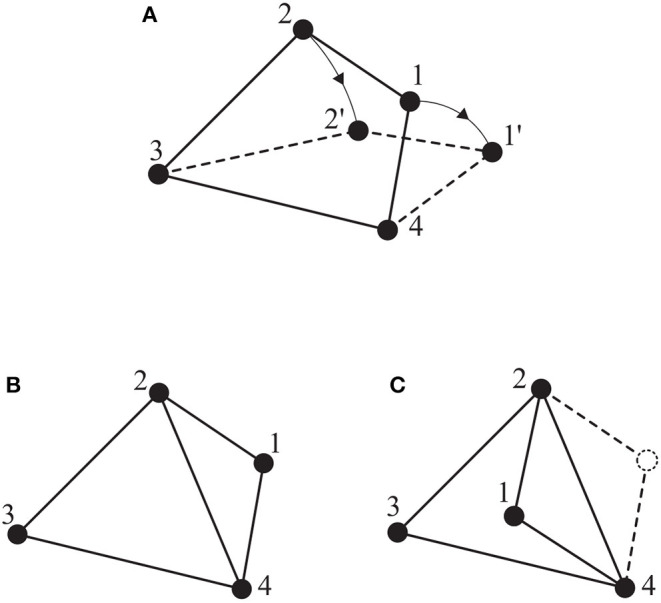
Several frameworks on ℜ^2^: **(A)** a non-rigid framework; **(B)** a minimally and infinitesimally rigid framework; **(C)** an equivalent but not consistent framework to **(B)**.

**Remark 1**. *It should be noted that similar to the aforementioned results hold on* ℜ^2^ *for a planar motion as well, e.g., a minimally and infinitesimally rigid framework with N* + 1 *vertices in* ℜ^2^ *has exactly* 2(*N* + 1) − 3 *edges (Cai and De Queiroz, 2015). Figure 3 illustrates the aforementioned concepts on* ℜ^2^.

### 3.3. Problem Formulation

In the leader-follower architecture that is adopted in this work, there is one global plan, i.e., a reference trajectory **p**_*d*_ (*t*) assigned only to the leader and several inter-agent distance specifications $d_{ij}^{⋆}$, (i,j)∈E to be satisfied in order to attain the desired formation. Therefore, under the assumption that the initial framework (G,p(0)) is minimally and infinitesimally rigid, we need to design decentralized control protocols to establish the desired formation and track the reference trajectory of the leader. Moreover, the only information employed in the control scheme should be acquired exclusively from the local navigation module of each vehicle and the distance/heading measurements among neighboring AUVs (i.e., no inter-agent communication is available).

**Remark 2**. *Under the assumption that the vehicle dynamics is fully actuated, the proposed control scheme shall output only forces expressed on the body frame of each vehicle. On the other hand, the rotational dynamics can be easily stabilized independently owing to their inherent passivity properties. Nevertheless, for a more energy efficient approach, the yaw motion can be controlled such that the longitudinal axis of the vehicles is aligned with the velocity vector that will be calculated by the proposed distance based control protocol, to reduce the hydrodynamic drag. Unfortunately, underwater vehicles that are unactuated in their translational motion, e.g., torpedo like vehicles where the sway and the heave DoFs are unactuated or vehicles with differential thrust configuration for which the sway motion is unactuated, are left open for future investigation owing to their inherent design complexity*.

## 4. Decentralized Control

Let us define the distance errors for each edge of the rigid graph, as:

(6) $$\begin{array}{r} e_{ij}\left(t\right)=∥{\text{p}}_{i}\left(t\right)-{\text{p}}_{j}\left(t\right)∥-d_{ij}^{⋆},\;\forall \left(i,j\right)\in E. \end{array}$$

A critical issue that has to be considered in multi-agent systems concerns collision avoidance among interacting agents. In this respect, the distance of the agents should be kept greater than a safety zone d$<d_{ij}^{⋆}$ to avoid collisions. Similarly, since the sensing devices have limited capabilities, it is also critical to retain neighboring agents close enough so that relative localization is available. Hence, the distance of the agents should be kept less than a sensing radius $\bar{d}>d_{ij}^{⋆}$ to secure the connectivity of the multi-agent system. Apparently, under the assumption that the initial condition satisfies the aforementioned collision avoidance and connectivity maintenance specifications, the control objective is to design a decentralized control protocol such that:

(7) $$\begin{array}{r} -{\underline{\rho }}_{ij}\left(t\right)<e_{ij}\left(t\right)<{\bar{\rho }}_{ij}\left(t\right),\;\forall \left(i,j\right)\in E \end{array}$$

for all *t* ≥ 0, where ρ_*ij*_*(t)*, ${\bar{\rho }}_{ij}\left(t\right)$ denote strictly positive and decreasing performance functions (Bechlioulis and Rovithakis, 2008) that satisfy $\underset{t\to \infty }{\lim}{\underline{\rho }}_{ij}\left(t\right)≜{\underline{\rho }}_{ij}^{\infty }>0$ and $\underset{t\to \infty }{\lim}{\bar{\rho }}_{ij}\left(t\right)≜{\bar{\rho }}_{ij}^{\infty }>0$, respectively. Notice that if we select ${\underline{\rho }}_{ij}\left(0\right)=d_{ij}^{⋆}-\underline{d}$ and ${\bar{\rho }}_{ij}\left(0\right)=\bar{d}-d_{ij}^{⋆}$, then satisfying (4) for all time guarantees collision avoidance and connectivity maintenance owing to the decreasing property of the performance functions ${\underline{\rho }}_{ij}\left(t\right),\;{\bar{\rho }}_{ij}\left(t\right)$. Moreover, selecting appropriately the decreasing rate and the steady state value of the performance functions ${\underline{\rho }}_{ij}\left(t\right),\;{\bar{\rho }}_{ij}\left(t\right)$ enforces transient and steady state performance specifications on the corresponding distance errors *e*_*ij*_(*t*). For instance, we could adopt exponentially decaying performance functions of the form ${\underline{\rho }}_{ij}\left(t\right)=\left(d_{ij}^{⋆}-\underline{d}-\rho_{\infty }\right)\exp\left(-\lambda t\right)+\rho_{\infty }$ and ${\bar{\rho }}_{ij}\left(t\right)=\left(\underline{d}-d_{ij}^{⋆}-\rho_{\infty }\right)\exp\left(-\lambda t\right)+\rho_{\infty }$, where λ and ρ_∞_ correspond to the decaying rate and the maximum error at the steady state, respectively.

**Remark 3**. *Distance based formation control, which has been extensively studied recently [for more details refer to the survey paper by Oh et al. (2015)], employs typically gradient based control laws, derived from quadratic potential functions of the distance errors, to establish the desired formation. Apparently, a similar to (3) rigidity matrix emerges during the calculation of the gradient, which verifies the resemblance with our approach. Nevertheless, notice that in our case, we extend the current state of the art by guaranteeing further predefined transient and steady state performance specifications, which also ensure collision avoidance and connectivity maintenance (i.e., relative localization), both of which are of utmost importance for the safe operation of the multi-agent system*.

### 4.1. Control Design

The proposed control protocol is first derived at the kinematic level assuming that the control signals are the linear body velocities. Subsequently, the kinematic controller is extended to the dynamic model, considering the actual control input signals, i.e., body forces. Hence, given a smooth and bounded desired trajectory ${\text{p}}_{d}\left(t\right)={\left[x_{d}\left(t\right),y_{d}\left(t\right),z_{d}\left(t\right)\right]}^{T}$ with bounded time derivatives, a priori known only to the leader, and any initial configuration close to the desired formation, that satisfies the collision avoidance and connectivity maintenance specifications, the control design proceeds as follows:

**Step 1:** Select the performance functions ${\underline{\rho }}_{ij}\left(t\right),\;{\bar{\rho }}_{ij}\left(t\right),\;\forall \left(i,j\right)\in E$ as:

(8) $$\begin{array}{r} {\underline{\rho }}_{ij}\left(t\right)=\left(d_{ij}^{⋆}-\underline{d}-\rho_{\infty }\right)\exp\left(-\lambda t\right)+\rho_{\infty } \end{array}$$

(9) $$\begin{array}{r} {\bar{\rho }}_{ij}\left(t\right)=\left(\underline{d}-d_{ij}^{⋆}-\rho_{\infty }\right)\exp\left(-\lambda t\right)+\rho_{\infty } \end{array}$$

to incorporate via the appropriate selection of ρ_∞_ and λ the desired performance specifications regarding the steady state error and the speed of convergence.

**Step 2:** Design the following velocity profile expressed in the inertial frame:

(10) $$\begin{array}{r} {\text{v}}_{I}=-k_{E}R^{T}\Xi_{E}E+I_{L}\left(-k_{P}\left({\text{p}}_{0}\left(t\right)-{\text{p}}_{d}\left(t\right)\right)+{\overset{∙}{\text{p}}}_{d}\left(t\right)\right),k_{E},k_{P}>0 \end{array}$$

where *R* ∈ ℜ^*l* × 3(*N* + 1)^ denotes the rigidity matrix defined in (3), $I_{L}≜{\left[{\text{I}}_{3\times 3},0_{3\times 3},\ldots ,0_{3\times 3}\right]}^{T}\in {ℜ}^{3\left(N+1\right)\times 3}$, *E* is the vector of modulated errors defined as:

(11) $$\begin{array}{r} E≜\text{col}{\left(\ln\left(\frac{1+\frac{e_{ij}\left(t\right)}{{\underline{\rho }}_{ij}\left(t\right)}}{1-\frac{e_{ij}\left(t\right)}{{\bar{\rho }}_{ij}\left(t\right)}}\right)\right)}_{\left(i,j\right)\in E}\in {ℜ}^{l} \end{array}$$

and Ξ_*E*_ denotes the diagonal matrix of the derivatives of the modulated errors with respect to the actual distance errors:

(12) $$\begin{array}{r} \Xi_{E}≜\frac{\partial E}{\partial e_{ij}}=\text{diag}{\left(\frac{{\underline{\rho }}_{ij}\left(t\right)+{\bar{\rho }}_{ij}\left(t\right)}{\left({\underline{\rho }}_{ij}\left(t\right)+e_{ij}\left(t\right)\right)\left({\bar{\rho }}_{ij}\left(t\right)-e_{ij}\left(t\right)\right)}\right)}_{\left(i,j\right)\in E}\in {ℜ}^{l\times l}. \end{array}$$

**Step 3:** Express the aforementioned velocity profile in the body frame of each vehicle as:

(13) $$\begin{array}{r} {\text{v}}_{d}≜\left[{\text{v}}_{0d}^{T},{\text{v}}_{1d}^{T},\ldots ,{\text{v}}_{Nd}^{T}\right]={\left(J_{\text{v}}\right)}^{-1}{\text{v}}_{I}, \end{array}$$

where $J_{\text{v}}≜\text{blockdiag}\left(J_{i}^{\text{v}}{\left(q_{i}\right)}_{i=0,1,\ldots ,N}\right)\in {ℜ}^{3\left(N+1\right)\times 3\left(N+1\right)}$.

**Step 4:** Define the velocity errors at the body frame:

(14) $$\begin{array}{r} {\tilde{\text{v}}}_{i}≜{\left[{\tilde{\text{v}}}_{i}^{u},{\tilde{\text{v}}}_{i}^{v},{\tilde{\text{v}}}_{i}^{w}\right]}^{T}={\text{v}}_{i}-{\text{v}}_{id},i=0,\ldots ,N \end{array}$$

and select the corresponding, exponential decaying, velocity performance functions $\rho_{{\text{v}}_{i}}\left(t\right)≜{\left[\rho_{{\text{v}}_{i}}^{u}\left(t\right),\rho_{{\text{v}}_{i}}^{v}\left(t\right),\rho_{{\text{v}}_{i}}^{w}\left(t\right)\right]}^{T}$ such that $|{\tilde{\text{v}}}_{i}^{u}\left(0\right)|<\rho_{{\text{v}}_{i}}^{u}\left(0\right)$, $|{\tilde{\text{v}}}_{i}^{v}\left(0\right)|<\rho_{{\text{v}}_{i}}^{v}\left(0\right)$, $|{\tilde{\text{v}}}_{i}^{w}\left(0\right)|<\rho_{{\text{v}}_{i}}^{w}\left(0\right)$ and $\underset{t\to \infty }{\lim}\rho_{{\text{v}}_{i}}^{u}\left(t\right)≜\rho_{{\text{v}}_{i}}^{u\infty }>0$, $\underset{t\to \infty }{\lim}\rho_{{\text{v}}_{i}}^{v}\left(t\right)≜\rho_{{\text{v}}_{i}}^{v\infty }>0$, $\underset{t\to \infty }{\lim}\rho_{{\text{v}}_{i}}^{w}\left(t\right)≜\rho_{{\text{v}}_{i}}^{w\infty }>0$, *i* = 0, 1, …, *N*.

**Step 5:** Finally, design the control law:

(15) $$\begin{array}{r} \left[\begin{array}{c} \tau_{u_{i}}\\ \tau_{v_{i}}\\ \tau_{w_{i}} \end{array}\right]=-k_{V}\Xi_{E_{{\text{v}}_{i}}}E_{{\text{v}}_{i}},k_{V}>0,i=0,1,\ldots ,N. \end{array}$$

where *E*_**v**_*i*__ denotes the vector of modulated velocity errors defined as:

(16) $$\begin{array}{r} E_{{\text{v}}_{i}}≜\frac{1}{2}{\left[\ln\left(\frac{1+\frac{{\tilde{\text{v}}}_{i}^{u}\left(t\right)}{\rho_{{\text{v}}_{i}}^{u}\left(t\right)}}{1-\frac{{\tilde{\text{v}}}_{i}^{u}\left(t\right)}{\rho_{{\text{v}}_{i}}^{u}\left(t\right)}}\right),\ln\left(\frac{1+\frac{{\tilde{\text{v}}}_{i}^{v}\left(t\right)}{\rho_{{\text{v}}_{i}}^{v}\left(t\right)}}{1-\frac{{\tilde{\text{v}}}_{i}^{v}\left(t\right)}{\rho_{{\text{v}}_{i}}^{v}\left(t\right)}}\right),\ln\left(\frac{1+\frac{{\tilde{\text{v}}}_{i}^{w}\left(t\right)}{\rho_{{\text{v}}_{i}}^{w}\left(t\right)}}{1-\frac{{\tilde{\text{v}}}_{i}^{w}\left(t\right)}{\rho_{{\text{v}}_{i}}^{w}\left(t\right)}}\right)\right]}^{T} \end{array}$$

and Ξ_*E*_**v**_*i*___ is the diagonal matrix of the derivatives of the modulated velocity errors with respect to the actual velocity errors:

(17) $$\begin{array}{r} \Xi_{E_{{\text{v}}_{i}}}≜\text{diag}\left(\left[\begin{array}{c} \frac{\rho_{{\text{v}}_{i}}^{u}\left(t\right)}{\left(\rho_{{\text{v}}_{i}}^{u}\left(t\right)+{\tilde{\text{v}}}_{i}^{u}\left(t\right)\right)\left(\rho_{{\text{v}}_{i}}^{u}\left(t\right)-{\tilde{\text{v}}}_{i}^{u}\left(t\right)\right)}\\ \frac{\rho_{{\text{v}}_{i}}^{v}\left(t\right)}{\left(\rho_{{\text{v}}_{i}}^{v}\left(t\right)+{\tilde{\text{v}}}_{i}^{v}\left(t\right)\right)\left(\rho_{{\text{v}}_{i}}^{v}\left(t\right)-{\tilde{\text{v}}}_{i}^{v}\left(t\right)\right)}\\ \frac{\rho_{{\text{v}}_{i}}^{w}\left(t\right)}{\left(\rho_{{\text{v}}_{i}}^{w}\left(t\right)+{\tilde{\text{v}}}_{i}^{w}\left(t\right)\right)\left(\rho_{{\text{v}}_{i}}^{w}\left(t\right)-{\tilde{\text{v}}}_{i}^{w}\left(t\right)\right)} \end{array}\right]\right). \end{array}$$

**Remark 4**. *Based on the assumption that the initial configuration of the multi-agent system meets the collision avoidance and connectivity maintenance specifications, the proposed error transformation (see **Step 2** and **5** of the aforementioned design process) guarantees that the boundedness of the modulated errors is simply sufficient to establish the control objective via (4). Notice that the logarithmic functions that are adopted to modulate the distance and velocity errors operate similarly to the barrier functions in constrained optimization, admitting high values when the control objectives tend to be violated; eventually preventing the distance and velocity errors from reaching the corresponding performance bounds. Consequently, collision avoidance and connectivity maintenance is secured analytically via the appropriate selection of the distance error performance bounds*.

**Remark 5**. *The aforementioned design process is decentralized in the sense that each vehicle requires the relative position of its neighbors and its own velocity only, which can be easily acquired by the onboard sensors without necessitating for network communication. Hence, the control protocol is independent of the global coordinate frame and does not require the local coordinate frames of all vehicles to be aligned*.

**Remark 6**. *The transient and steady state response as well as the collision avoidance and connectivity maintenance specifications are encapsulated in the proposed control protocol via the appropriate selection of the performance functions $\rho_{ij}\left(t\right),\;\forall \left(i,j\right)\in E$. In addition, the velocity performance functions ρ*_**v**_*i*__*(*t*), *i* = 0, 1, …, *N* impose prescribed performance on the body velocity errors ${\tilde{\text{v}}}_{i},i=0,1,\ldots ,N$. However, it should be stressed that although such performance specifications are not required, their selection (the only hard constraint attached to their definition is related to their initial value, see **Step 4**) affects the evolution of the distance errors within the corresponding performance envelopes. Similarly, the selection of the control gains *k*_*E*_, *k*_*V*_ affects the control input characteristics as well. Decreasing the gain values leads to increased oscillatory behavior within the performance envelopes, which can be suppressed when adopting higher gain values enlarging however the control effort both in magnitude and rate. Apparently, fine tuning might be needed during real-time implementation to meet certain input constraints that affect severely the AUVs dynamics*.

### 4.2. Stability Analysis

The following theorem summarizes the main results of this work.

**Theorem 1**. *Consider a group of N* + 1 *AUVs obeying the dynamics (1), at an initial minimally and infinitesimally rigid configuration. The decentralized control protocol proposed in subsection 4.1 guarantees that the leader tracks the reference trajectory and the desired formation is attained with prescribed transient and steady state performance, while avoiding inter-agent collisions and connectivity breaks*.

*Proof*. The proof proceeds similarly to Bechlioulis et al. (2018), thus a brief sketch will be given herein. Particularly, based on Theorem 54 (pp. 476) in Sontag (1998) and given that the initial distance and velocity errors satisfy by construction $-{\underline{\rho }}_{ij}\left(0\right)<e_{ij}\left(0\right)<{\bar{\rho }}_{ij}\left(0\right),\;\forall j\in N_{i}$ and $|{\tilde{\text{v}}}_{i}^{u}\left(0\right)|<\rho_{{\text{v}}_{i}}^{u}\left(0\right)$, $|{\tilde{\text{v}}}_{i}^{v}\left(0\right)|<\rho_{{\text{v}}_{i}}^{v}\left(0\right)$, $|{\tilde{\text{v}}}_{i}^{w}\left(0\right)|<\rho_{{\text{v}}_{i}}^{w}\left(0\right)$, respectively for all *i* = 0, 1, …, *N* as well as that certain continuity and integrability conditions of the closed loop system are upheld, there exists a maximal interval [0, τ_*f*_) with $\tau_{f}\in \left\{{ℜ}_{+}^{*},\infty \right\}$, on which $-{\underline{\rho }}_{ij}\left(t\right)<e_{ij}\left(t\right)<{\bar{\rho }}_{ij}\left(t\right),\;\forall j\in N_{i}$ and $|{\tilde{\text{v}}}_{i}^{u}\left(t\right)|<\rho_{{\text{v}}_{i}}^{u}\left(t\right)$, $|{\tilde{\text{v}}}_{i}^{v}\left(t\right)|<\rho_{{\text{v}}_{i}}^{v}\left(t\right)$, $|{\tilde{\text{v}}}_{i}^{w}\left(t\right)|<\rho_{{\text{v}}_{i}}^{w}\left(t\right)$, respectively, for all *i* = 0, 1, …, *N* and all *t* ∈ [0, τ_*f*_). Therefore, the modulated distance and velocity error vectors *E* and *E*_**v**_*i*__, *i* = 0, 1, …, *N* are well-defined for all *t* ∈ [0, τ_*f*_). Subsequently, let us assume that τ_*f*_ ≠ ∞ (otherwise the problem would be trivially solved, since the inequalities would hold for all time). In the sequel, following standard Lyapunov arguments, we shall prove that, for all *t* ∈ [0, τ_*f*_), the distance and velocity errors will evolve strictly within the corresponding upper and lower bounds dictated by the performance functions irrespectively of τ_*f*_. Thus, invoking Proposition C.3.6 (pp. 481) in Sontag (1998), it can be proved by contradiction that τ_*f*_ = ∞.

Hence, consider a candidate Lyapunov function of the modulated distance errors *E* as follows:

(18) $$\begin{array}{r} V=\frac{1}{2}E^{T}E. \end{array}$$

Differentiating with respect to time, we obtain:

(19) $$\begin{array}{r} \overset{∙}{V}=E^{T}\frac{\partial E}{\partial e_{ij}}\text{col}\left({ė}_{ij}\left(t\right)\right)+E^{T}\frac{\partial E}{\partial {\bar{\rho }}_{ij}}\text{col}\left({\overset{∙}{\bar{\rho }}}_{ij}\left(t\right)\right)+E^{T}\frac{\partial E}{\partial {\underline{\rho }}_{ij}}\text{col}\left({\overset{∙}{\underline{\rho }}}_{ij}\left(t\right)\right). \end{array}$$

Employing the fact that $\frac{\partial E}{\partial e_{ij}}≜\Xi_{E}$, $\frac{\partial E}{\partial {\bar{\rho }}_{ij}}≜\Xi_{E}\Xi_{{\bar{\rho }}_{ij}}$, $\frac{\partial E}{\partial {\underline{\rho }}_{ij}}≜\Xi_{E}\Xi_{{\underline{\rho }}_{ij}}$ for some bounded diagonal matrices Ξ_ρ_*ij*__, $\Xi_{{\bar{\rho }}_{ij}}$ and **col**(ė_*ij*_(*t*)) = *RJ*_**v**_**v**, where *R* denotes the rigidity matrix and $\text{v}≜{\left[{\text{v}}_{0}^{T},{\text{v}}_{1}^{T},\ldots ,{\text{v}}_{N}^{T}\right]}^{T}$ is the overall vector of the body velocities of the AUVs, and adding and subtracting the term $E^{T}\Xi_{E}R{\text{v}}_{I}$, we arrive at:

(20) $$\begin{array}{l} \dot{V}=E^{T}\Xi_{E}Rv_{I}+E^{T}\Xi_{E}RJ_{v}\tilde{v}+E^{T}\Xi_{E}(\Xi_{{\bar{\rho }}_{ij}}col\left({\dot{\bar{\rho }}}_{ij}(t)\right)\\ \;+\Xi_{\rho_{ij}}col\left({\dot{\underline{\rho }}}_{ij}(t)\right)) \end{array}$$

where $\tilde{\text{v}}≜{\left[{\tilde{\text{v}}}_{0}^{T},{\tilde{\text{v}}}_{1}^{T},\ldots ,{\tilde{\text{v}}}_{N}^{T}\right]}^{T}$. Substituting the control signal (10) and utilizing the fact that ${\tilde{\text{v}}}_{i}=\text{diag}\left(\rho_{{\text{v}}_{i}}\left(t\right)\right)\text{tanh}\left(E_{{\text{v}}_{i}}\right)$, where *E*_**v**_*i*__ denote the modulated velocity errors, we obtain:

(21) $$\begin{array}{l} \dot{V}=-k_{E}E^{T}\Xi_{E}RR^{T}\Xi_{E}E+E^{T}\Xi_{E}(RJ_{v}col\left(\rho_{v_{i}}(t)tanh(E_{v_{i}})\right)\\ \;-RI_{L}(-k_{P}(p_{0}(t)-p_{d}(t))+{\dot{p}}_{d}(t))\\ \;+\Xi_{{\bar{\rho }}_{ij}}col\left({\dot{\bar{\rho }}}_{ij}(t)\right)+\Xi_{{\underline{\rho }}_{ij}}col\left({\dot{\underline{\rho }}}_{ij}(t)\right)). \end{array}$$

It should be noted that: (a) the rigidity matrix *R* is bounded by definition since it is formed by normalized vectors as in (4), (b) the Jacobian matrix is bounded by definition as it involves only sinusoidal terms, (c) the performance functions are bounded by construction, (d) the hyperbolic tangent function is bounded, (e) the reference trajectory of the leader is assumed bounded with bounded derivatives and (f) the derivative of the performance functions are bounded by construction. Therefore, there exists an upper bound of the right parenthesis in the second term, which is independent of τ_*f*_. Hence, invoking the positive definiteness of the square matrix *RR*^*T*^ by Lemma 2, it is easy to deduce the existence of a positive constant Ē, which depends of the aforementioned upper bound, such that $\overset{∙}{V}\leq 0$ for all ||*E*|| ≥ Ē. Thus, employing the Uniform Ultimate Boundedness Theorem (see Theorem 4.18 in Khalil, 2001), we derive the boundedness of all elements of the modulated error vector *E* as defined in (11), from which it is straightforward to deduce that (4) is strictly satisfied for all *t* ∈ [0, τ_*f*_) (see Remark 4). Moreover, since *E* was proven bounded then the velocity profiles **v**_*I*_ and consequently **v**_*d*_ and its derivative remain bounded as well. Similarly, invoking the aforementioned boundedness properties and observing the proportional and derivative terms of the leader's control law in (10), we also establish accurate tracking of the reference trajectory by the leader for all *t* ∈ [0, τ_*f*_) and for a sufficiently high gain *k*_*P*_. Finally, what remains to be proved is that $|{\tilde{\text{v}}}_{i}^{u}\left(t\right)|<\rho_{{\text{v}}_{i}}^{u}\left(t\right)$, $|{\tilde{\text{v}}}_{i}^{v}\left(t\right)|<\rho_{{\text{v}}_{i}}^{v}\left(t\right)$, $|{\tilde{\text{v}}}_{i}^{w}\left(t\right)|<\rho_{{\text{v}}_{i}}^{w}\left(t\right)$ are strictly satisfied for all *i* = 0, 1, …, *N* and all *t* ∈ [0, τ_*f*_). Hence, we follow the aforementioned line of proof for the velocity modulated errors *E*_**v**_*i*__, 0, 1, …, *N* by adopting the Lyapunov candidate function $V_{{\text{v}}_{i}}=\frac{1}{2}E_{{\text{v}}_{i}}^{T}E_{{\text{v}}_{i}}$ for each agent. Therefore, differentiating with respect to time and substituting the control law (15) and the AUV dynamics (1) (only the translational dynamics), we obtain:

(22) $$\begin{array}{l} {\dot{V}}_{v_{i}}=-k_{V}E_{v_{i}}^{T}\Xi_{E_{v_{i}}}M_{i}^{-1}\Xi_{E_{v_{i}}}E_{v_{i}}+E_{v_{i}}^{T}\Xi_{E_{v_{i}}}(M_{i}^{-1}(\tau_{E_{i}}-C_{i}(v_{i})v_{i}\\ \;-D_{i}(v_{i})v_{i}-g(\eta_{i}))-{\dot{v}}_{id}\\ \;-diag\left({\dot{\rho }}_{v_{i}}(t)\right)diag\left(\rho_{v_{i}}(t)\right)tanh(E_{v_{i}})). \end{array}$$

Similarly to the previous step, notice that all terms in the right parenthesis are bounded by construction or by assumption; hence invoking the positive definiteness of the inertia matrix **M**_*i*_, it is easy to conclude the boundedness of all elements of the modulated velocity errors *E*_**v**_*i*__, from which it is straightforward to deduce that $|{\tilde{\text{v}}}_{i}^{u}\left(t\right)|<\rho_{{\text{v}}_{i}}^{u}\left(t\right)$, $|{\tilde{\text{v}}}_{i}^{v}\left(t\right)|<\rho_{{\text{v}}_{i}}^{v}\left(t\right)$, $|{\tilde{\text{v}}}_{i}^{w}\left(t\right)|<\rho_{{\text{v}}_{i}}^{w}\left(t\right)$ are strictly satisfied for all *i* = 0, 1, …, *N* and all *t* ∈ [0, τ_*f*_). Moreover, since *E*_**v**_*i*__ was proven bounded then the control signals τ_*u*_*i*__, τ_*v*_*i*__ and τ_*w*_*i*__ remain bounded, which completes the proof.

**Remark 7**. *The proposed control protocol achieves its goals without resorting to the need of rendering the ultimate bounds of the modulated distance and velocity errors *E*, E*_**v**_*i*__ *arbitrarily small by adopting extreme values of the control gains *k*_*P*_, *k*_*E*_, and *k*_*V*_. In the same spirit, large model uncertainty and external disturbances involved in the vehicle non-linear model (1) can be compensated, as they affect only the size of the ultimate bound of *E*, E*_**v**_*i*__, *but leave unaltered the achieved stability properties. Since we do not consider input constraints in this work, notice that no matter how large the model uncertainty is [i.e., how large the ultimate bound of the modulated errors *E*, E*_**v**_*i*__, *extracted by (21) and (22), is] the actual performance given in (4), which is solely determined by the designer-specified performance functions $-{\underline{\rho }}_{ij}\left(t\right),\;{\bar{\rho }}_{ij}\left(t\right),\;\forall j\in N_{i}$, is isolated by the model uncertainties, thus extending greatly the robustness of the proposed control scheme*.

## 5. Simulation Results

We consider a leader-follower scheme composed of five identical underwater robotic vehicles. The model that was used for simulation is a 4 DoFs Seabotix LBV (LBV150, 2006), actuated in surge, sway, heave and yaw via a 4 thruster set configuration (the motion in roll and pitch has been neglected as both DoFs are passive and affect minimally the others). The main inertial and hydrodynamic parameters of Seabotix LBV150 are given in Table 1, as they were identified in Karras et al. (2018). The simulation framework was implemented in the Robot Operating System (ROS, 2009) using UWSim (Prats et al., 2012).

**Table 1:** The dynamic parameters of the Seabotix LBV150.

**Parameter**	**Value**	**Description**
$m_{x}=m+X_{\overset{∙}{u}}$	9.7532	Mass and added mass along surge axis
$m_{y}=m+Y_{\overset{∙}{v}}$	8.6636	Mass and added mass along sway axis
*m*_*z*_ = *m* + *Z*_ẇ_	10.898	Mass and added mass along heave axis
*I*_*z*_	0.1589	Inertia about yaw axis
*X*_*u*_	−8.6040	Linear damping term along surge axis
*Y*_*v*_	−18.1106	Linear damping term along sway axis
*Z*_*w*_	−17.1828	Linear damping term along heave axis
*N*_*r*_	−1.4146	Linear damping term about yaw axis
*X*_|*u*|*u*_	−17.8534	Quadratic damping term along surge axis
*Y*_|*v*|*v*_	−1.0594	Quadratic damping term along sway axis
*Z*_|*w*|*w*_	−3.6482	Quadratic damping term along heave axis
*N*_|*r*|*r*_	−10.3483	Quadratic damping term about yaw axis
*W* − *B*	−1.1881	Vehicle weight minus buoyancy

**Figure 4:**
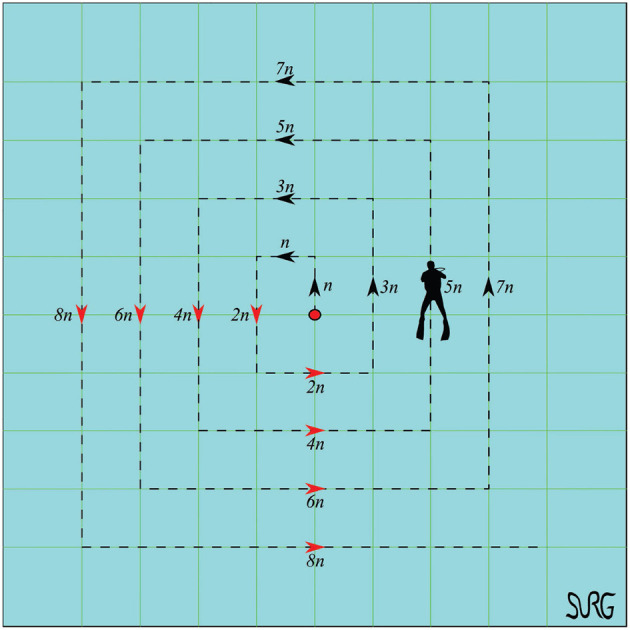
Compass box search pattern. In this work we selected *n* = 3 m.

**Figure 5:**
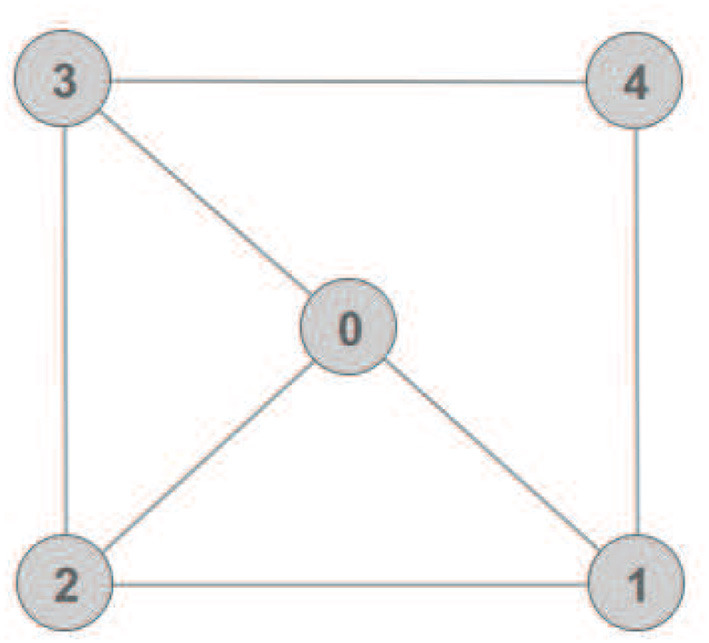
The adopted undirected graph with *l* = 7 edges, which is minimally rigid in ℜ^2^.

The task of scanning the sea-bed was assigned to the multi-agent system. The scan was performed according to a pattern, used by divers for object recovery, called “compass box search pattern” (see Figure 4). The aforementioned scanning trajectory that is expressed in the world frame is assigned exclusively to the leader, which acquires its absolute position with respect to the world frame via a USBL system. On the contrary, the followers are not required to have an absolute positioning system since their actual velocity command is directly calculated at their body fixed frames, since only relative distances and bearings among neighboring agents are employed. Moreover, a squared formation of edge 1 m (see Figure 5) was chosen with the leader (agent 0) positioned at the center and four other equidistant agents (agents 1–4) around it. It should be noted that the desired formation specifications were defined on the *x* − *y* plane, where the proposed scheme was applied (the orientation of the vehicles was controlled such that their longitudinal axis is aligned with the velocity vector that was calculated by the proposed distance based control scheme). Notice further that the aforementioned framework is minimally and infinitesimally rigid since the underlying graph G=({0,1,2,3,4},{(0,1),(0,2),(0,3),(1,2),(1,4),(2,3),(3,4)}) has exactly *l* = 2 × 5 − 3 = 7 edges (see Remark 1). On the other hand, the depth was stabilized close to 10 m by an independent fixed-gain PID controller. To make the simulation more realistic, ocean currents with slowly varying direction of speed within 0.1–0.3 m/s were considered. Finally, the cruising speed of the desired trajectory that mimics the “compass box search” was 0.5 m/s.

The collision avoidance and connectivity specifications were selected as: (a) sensing range $\bar{d}=4$ m and (b) safety distance *d* = 0.4 m. Moreover, it was requested to establish the formation with minimum convergence speed $\exp\left(-\frac{t}{5}\right)$ and retain it with maximum steady state error 0.1 m (i.e., the errors should reach close to zero with a slight deviation within 25 s). Hence, exponential performance functions were selected as in (8) and (9) to incorporate the aforementioned performance specifications. Similar performance functions were adopted for the velocity errors as well. Finally, the control gains were chosen as *k*_*E*_ = 1, *k*_*P*_ = 5, *k*_*V*_ = 100.

**Figure 6:**
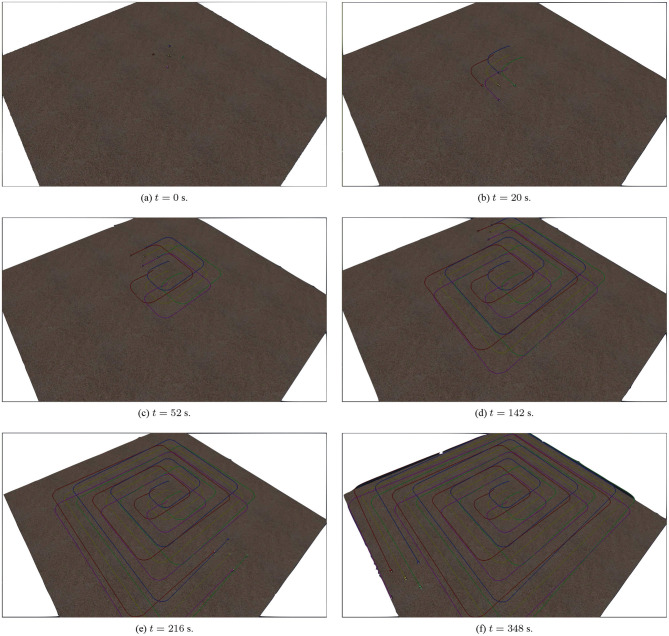
The evolution of the seabed scanning for six consecutive time instances.

**Figure 7:**
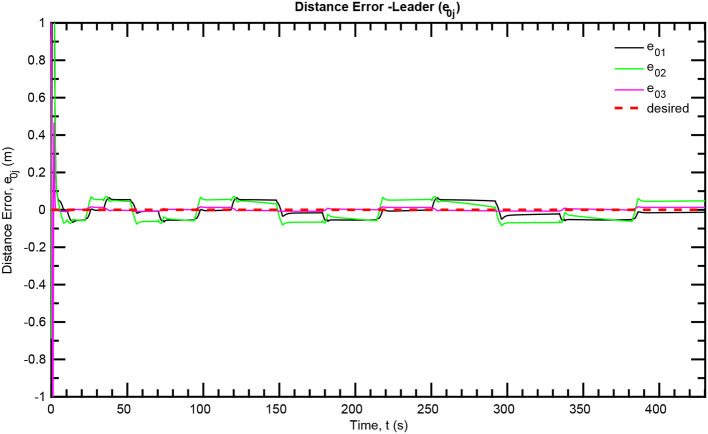
Distance errors—Leader.

**Figure 8:**
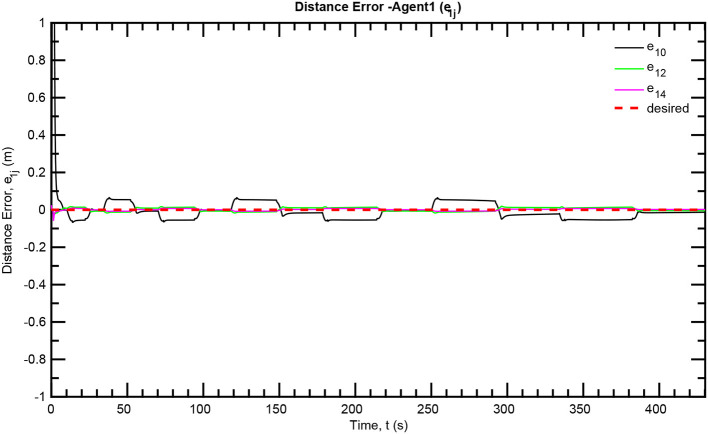
Distance errors—Follower 1.

**Figure 9:**
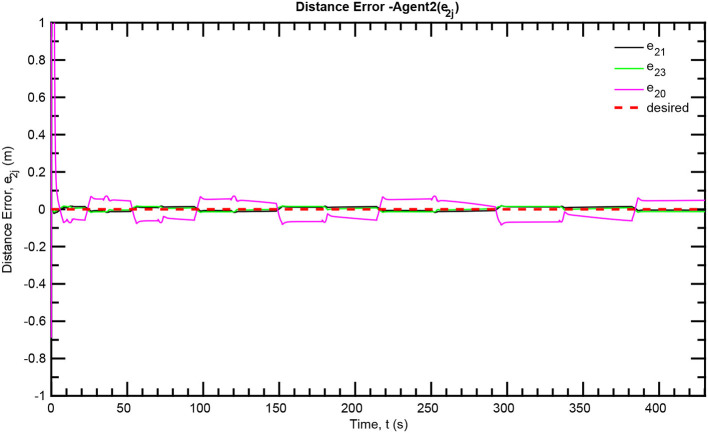
Distance errors—Follower 2.

**Figure 10:**
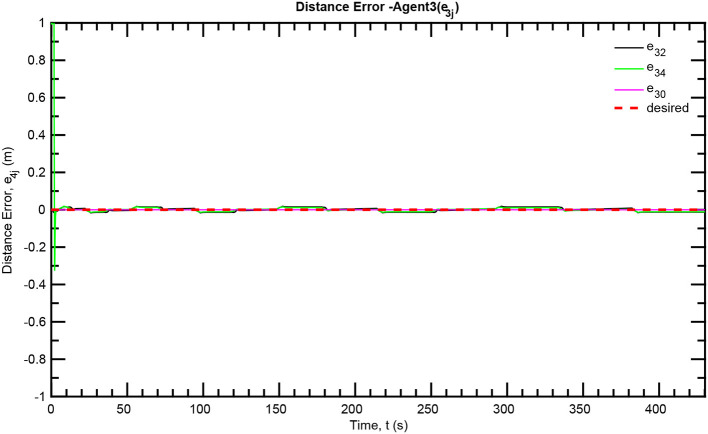
Distance errors—Follower 3.

**Figure 11:**
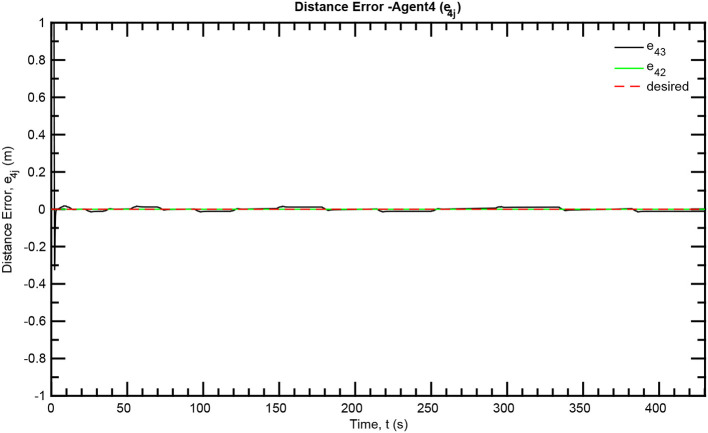
Distance errors—Follower 4.

**Figure 12:**
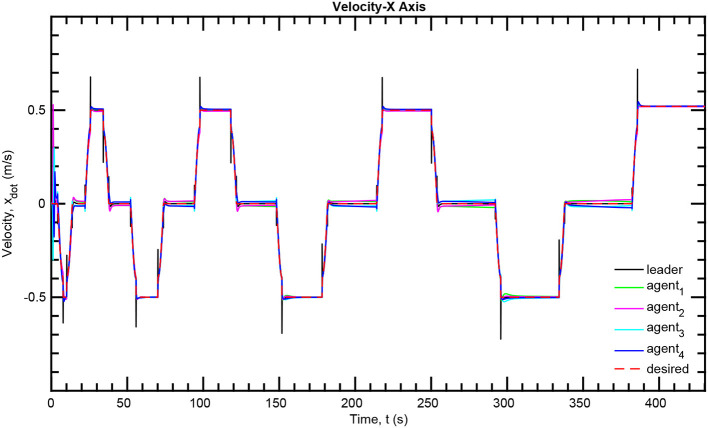
Velocity tracking—X axis.

**Figure 13:**
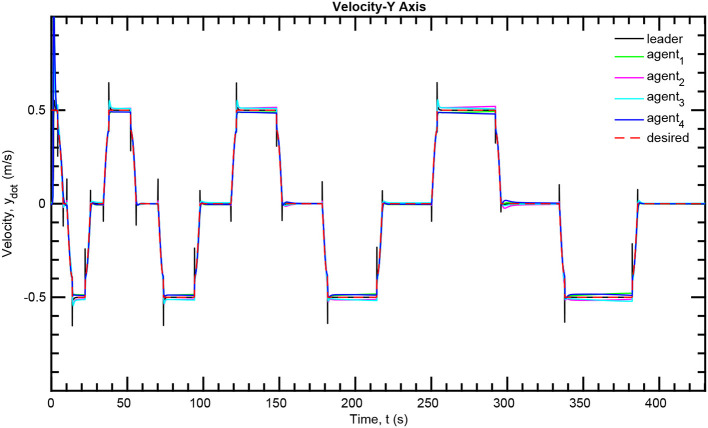
Velocity tracking—Y axis.

**Figure 14:**
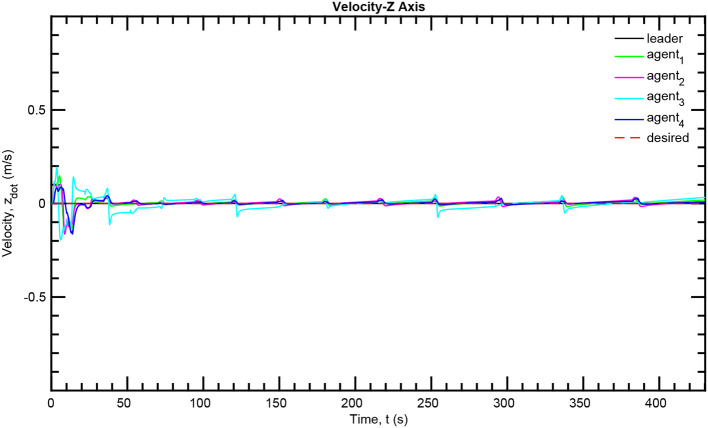
Velocity tracking–Z axis.

**Figure 15:**
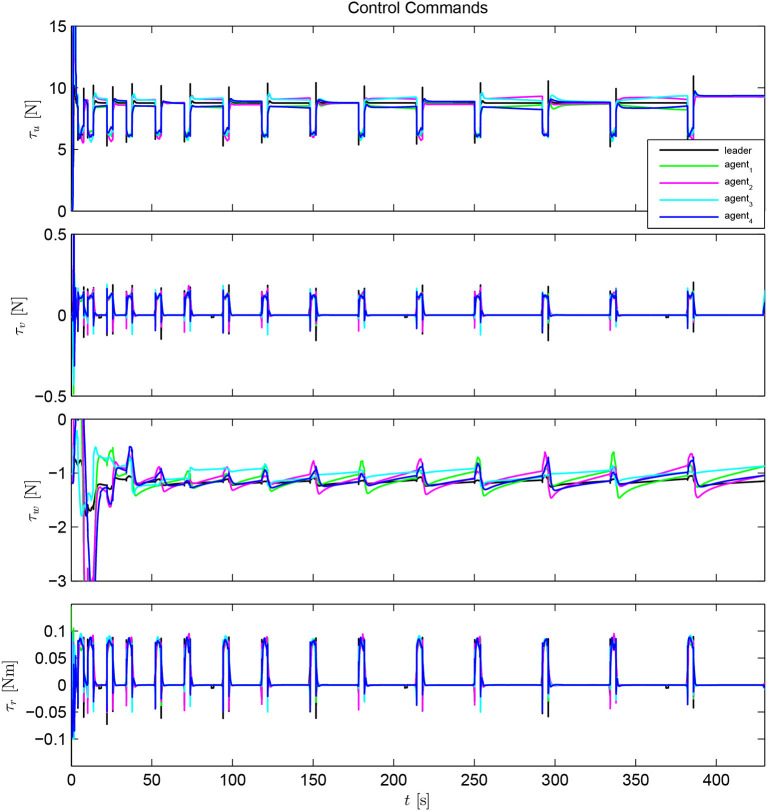
Control input signals.

The simulation results of the aforementioned study are illustrated below. The evolution of the sea-bed scanning, which is displayed in the Supplementary Video, is also depicted in Figure 6 for six consecutive time instants. Figures 7–11 illustrate the distance errors of each agent with respect to its neighbors. Notice that the inter-agent distance errors are kept <0.1 m, as determined by the selected performance functions. Similarly, Figures 12–14 depict the velocity of the agents expressed in X, Y, and Z axes of the global frame along with the desired velocity profile. Finally, the control input signals are depicted in Figure 15. Apparently, the whole formation follows the desired motion profile with fine accuracy (reasonable spikes appear at the corners of the motion profile). As it was predicted in the analysis, the formation control problem was safely tackled with predefined performance specifications and bounded signals, despite the external disturbances.

## 6. Discussion and Conclusions

This paper proposed a solution to the formation control problem for multiple AUVs in a leader-follower architecture. The derived control protocol guarantees formation establishment with prescribed transient and steady state performance while avoiding collisions and connectivity breaks and despite the presence of external disturbances and dynamic model uncertainty. Moreover, no explicit communication among the fleet is needed. Furthermore, for each AUV the control signal is calculated based only on the relative position of the neighboring vehicles and its own velocity, which both can be easily acquired by the onboard sensors. Additionally, the proposed decentralized control protocol is of low complexity. Finally, realistic simulation results clarified and verified the proposed approach.

Future research directions will be devoted toward studying the effect of: (i) underactuated translational dynamics (i.e., AUVs unactuated in the sway or heave degrees of freedom), (ii) input uncertainties (i.e., mapping uncertainties between desired body forces/torques and actuator commands) and constraints (e.g., the thruster limitations) as well as (iii) sensor filtering (underwater relative localization is plagued with noise, intermittent failures and latency) on the closed loop stability, in order to increase the applicability of the proposed control methodology in open sea scenarios. Extra attention should also be pledged on studying how the achieved transient and steady state performance specifications could encapsulate further configuration constraints that may arise owing to extra sector-based (not only range-based) limited capabilities of the on board sensors (e.g., the limited field of view of cameras or sonars) adopted to acquire relative localization. Finally, the increasingly challenging mission scenarios in the field of marine robotics, call for inexpensive and robust control solutions for obstacle avoidance.

## Author Contributions

CB took over the technical writing of the manuscript. FG and GK conducted the simulation study. Finally, KK supervised the work.

### Conflict of Interest

The authors declare that the research was conducted in the absence of any commercial or financial relationships that could be construed as a potential conflict of interest.

## Footnotes

^1^Although underwater robots are equipped with acoustic modems to communicate with the surface control station, avoiding the use of intense explicit inter-robot communication is clearly motivated by the limited bandwidth of underwater acoustic devices and issues related to time delays and packet drops, which are critical in such communication medium.

## Supplementary Material

Supplementary Video Scanning the seabed via the compass box search pattern.
